# Does responsiveness to arbuscular mycorrhizal fungi depend on plant invasive status?

**DOI:** 10.1002/ece3.3226

**Published:** 2017-07-10

**Authors:** Kurt O. Reinhart, Ylva Lekberg, John Klironomos, Hafiz Maherali

**Affiliations:** ^1^ Fort Keogh Livestock & Range Research Laboratory United States Department of Agriculture‐ Agricultural Research Service Miles City MT USA; ^2^ MPG Ranch and Ecosystem and Conservation Sciences University of Montana Missoula MT USA; ^3^ Department of Biology University of British Columbia ‐ Okanagan Campus Kelowna BC Canada; ^4^ Department of Integrative Biology University of Guelph Guelph ON Canada

**Keywords:** arbuscular mycorrhizal fungi, grassland, invasion ecology, mixed‐grass prairie, mycorrhizal responsiveness, phylogenetic signal, phylogeny, tallgrass prairie

## Abstract

Differences in the direction and degree to which invasive alien and native plants are influenced by mycorrhizal associations could indicate a general mechanism of plant invasion, but whether or not such differences exist is unclear. Here, we tested whether mycorrhizal responsiveness varies by plant invasive status while controlling for phylogenetic relatedness among plants with two large grassland datasets. Mycorrhizal responsiveness was measured for 68 taxa from the Northern Plains, and data for 95 taxa from the Central Plains were included. Nineteen percent of taxa from the Northern Plains had greater total biomass with mycorrhizas while 61% of taxa from the Central Plains responded positively. For the Northern Plains taxa, measurable effects often depended on the response variable (i.e., total biomass, shoot biomass, and root mass ratio) suggesting varied resource allocation strategies when roots are colonized by arbuscular mycorrhizal fungi. In both datasets, invasive status was nonrandomly distributed on the phylogeny. Invasive taxa were mainly from two clades, that is, Poaceae and Asteraceae families. In contrast, mycorrhizal responsiveness was randomly distributed over the phylogeny for taxa from the Northern Plains, but nonrandomly distributed for taxa from the Central Plains. After controlling for phylogenetic similarity, we found no evidence that invasive taxa responded differently to mycorrhizas than other taxa. Although it is possible that mycorrhizal responsiveness contributes to invasiveness in particular species, we find no evidence that invasiveness in general is associated with the degree of mycorrhizal responsiveness. However, mycorrhizal responsiveness among species grown under common conditions was highly variable, and more work is needed to determine the causes of this variation.

## 1 INTRODUCTION

Many natural plant communities are either invaded or at risk of invasion by non‐native plant species (Pimentel, Lach, Zuniga, & Morrison, 2000). The negative consequences of such invasions have stimulated extensive research aimed at identifying the responsible mechanisms, so as to identify efficacious control/management strategies (Mack et al., 2000). Although several mechanisms are likely in play (Gurevitch, Fox, Wardle, Inderjit, & Taub, 2011), exotic species are generally thought to be less reliant on *specialist* mutualists than native taxa (Richardson, Allsopp, D'Antonio, Milton, & Rejmanek, 2000; Vogelsang & Bever, 2009) but whether this also extends to *generalist* mutualists is less known. Arbuscular mycorrhizal fungi (AMF) are obligate biotrophs that colonize about 75% of all plant species (Brundrett, 2009) with apparent low specificity (Lekberg & Waller, 2016). These ubiquitous fungi can provide increased nutrient uptake, drought tolerance, and pathogen protection to plants in return for carbon (Smith & Read, 2008). AMF could contribute to invasiveness if exotic plants benefitted more from these services than native plants, or if exotic plants were less dependent on AMF and/or could disrupt this mutualism in native plants. To determine whether exotic plants are less reliant on the mutualism with AMF, an important first step is to compare the mycorrhizal status and responsiveness (measured as the biomass difference between inoculated plants and non‐inoculated controls; Janos, 2007) of native and exotic plants.

Some have suggested that a weak mycorrhizal responsiveness may be a general mechanism of plant invasion (van der Putten, Klironomos, & Wardle, 2007; Vogelsang & Bever, 2009) because invasions often occur in disturbed habitats (Mooney & Hobbs, 2000) that tend to harbor lower AMF abundance (Abbott & Robson, 1991). Consistent with this hypothesis is the fact that some well‐studied noxious invaders in North America are either nonmycorrhizal or have a low mycorrhizal responsiveness (e.g., Busby, Gebhart, Stromberger, Meiman, & Paschke, 2011; Stinson et al., 2006) and that exotic plants in California are disproportionally from nonmycorrhizal families (Pringle et al., 2009). Also, careful comparisons of native and exotic populations of both St. John's Wort (Hypericum perforatum; Seifert, Bever, & Maron, 2009) and yellow star thistle (Centaurea solstilialis; Waller, Callaway, Klironomos, Ortega, & Maron, 2016) have documented more ruderal traits and a reduced AMF responsiveness in the exotic populations.

On the other hand, AMF can increase growth and competitiveness of spotted knapweed (Centaurea stoebe; Marler, Zabinski, & Callaway, 1999), which is one of the most invasive weeds in the intermountain west of the USA. This and another invasive forb (*Euphorbia esula*) can also increase AMF abundance and diversity compared to remnant native communities (Lekberg, Gibbons, Rosendahl, & Ramsey, 2013). And, contrary to California grasslands, exotic plants in Great Britain and Germany were more likely to be from families that were more dependent on AMF (i.e., greater proportion that were obligatory mycorrhizal and lower proportion that were facultatively mycorrhizal) than native plants (Hempel et al., 2013; Pringle et al., 2009). Thus, it is clear that interactions with AMF differ drastically among invasive plants and possibly across regions being invaded.

A recent systematic literature search and summary (Bunn, Ramsey, & Lekberg, 2015) reported no appreciable difference between invasive and native plants in their growth responses to AMF, suggesting that invasions do not select for directional shifts in AMF associations. While this meta‐analysis represents the most comprehensive summary of published studies to date, there are some important limitations to the inferences drawn from such analyses. Specifically, meta‐analyses cannot always account for a high level of experimental heterogeneity among the included studies. The Bunn et al. (2015) analysis, for example, included data for 55 invasive and 70 native species from 67 studies that varied in experimental conditions (e.g., soil chemistry, identity of AMF taxa, growth conditions), which are known to affect plant–AMF interactions (Johnson, Wilson, Bowker, Wilson, & Miller, 2010; Klironomos, 2003; Stahl & Smith, 1984). These experimental nuances are not easily incorporated into meta‐analysis as factors or covariates (Koricheva, Gurevitch, & Mengersen, 2013) and may have prevented detection of actual differences between invasive non‐native vs. native taxa that are apparent only with a dataset obtained from a common set of experimental conditions. Also, plant responses to AMF are often phylogenetically conserved (Anacker, Klironomos, Maherali, Reinhart, & Strauss, 2014; Reinhart, Wilson, & Rinella, 2012), and phylogenetic nonindependence can bias results if the pool of species in comparison groups is disproportionately from clades that are highly responsive (or unresponsive) to AMF.

To circumvent these limitations, we analyzed two relatively large datasets that approximated the total sample size of the Bunn et al. (2015) meta‐analysis where plants were grown under the same environmental conditions and where the effects of shared ancestry were controlled. We generated new mycorrhizal responsiveness data for 68 plant species from the Northern Plains (14 invasive: 54 noninvasive) and performed separate tests for a published dataset with 95 taxa (11 invasive: 84 noninvasive) from the Central Plains (Wilson & Hartnett, 1998). A previous analysis of the Central Plains dataset which did not control for phylogeny found that native grassland plants were more responsive to AMF than taxa from outside the region (fig. 3 in Pringle et al., 2009), and we tested whether the same result would be found if the effects of shared ancestry were included.

## 2 METHODS

### 2.1 Northern Plains mycorrhizal growth response experiment

AMF spores were originally derived by either extraction from field soil or trap cultures of plant root (or soil) samples from four sites in Custer County, Montana, USA. The spores were extracted using wet sieving and sucrose density gradient centrifugation (Brundrett, Melville, & Peterson, 1994). Spores were extracted into a water solution. To propagate the fungal inoculum, we used *Sorghum sudanese* as the plant host. Seeds of *S. sudanese* were surface sterilized (placed in 10% bleach solution for 10 min then thoroughly rinsed) and then sowed into aluminum trays with once‐autoclaved vermiculite. Eighteen pots (3.21 L) were planted with three 1‐week‐old *S. sudanese* seedlings and inoculated with water containing AMF spores. There were nine AMF morphotypes identified, and 8–50 AMF spores were used to inoculate two replicate pots per morphotype. Eighteen additional control pots with the same number of *S. sudanese* seedlings were sham inoculated (Tintjer & Rudgers, 2006) with aliquots of the microbial solution from which AMF spores had been aspirated. The sham inoculant was necessary to control for additional forms of microbial life (e.g., bacteria, saprobic fungi) in the water solution containing AMF spores. Pots were filled with once autoclaved field soil (Eapa fine loam, frigid Aridic Argiustolls) from a calcareous grassland site. Pots were watered three times a week for 6 months. At harvest, we discarded shoots and the top 1 cm of soil. The remaining soil, which was to be used as inoculant for the mycorrhizal growth response experiment, was homogenized (i.e., one AMF mixture and one control mixture) and roots were diced into 1 cm fragments on 6 February 2014. Experimental units (pots) were prepared on 7 February 2014 (details below). We note that the AMF inoculum, although representative of a mycorrhizal community for the Northern Plains, still represents a simplification of the many AMF communities in the region (Reinhart & Anacker, 2014).

To determine the invasive and noninvasive species to be used in the experiment, we consulted regional experts. Invasive status was based on classifications by the US Forest Service (http://www.fs.fed.us/database/feis/plants/weed/index.html). Although the species sampled included more noninvasive than invasive species, this ratio reflects the scarcity of invasive relative to noninvasive taxa in the flora. This ratio also permits an unbiased estimate of phylogenetic effects on variation in plant growth response to mycorrhizal inoculation. Seed for the 68 selected species was collected by the authors from field sites or donated by other researchers. However, cases of insufficient seed, low germination rates, or both, necessitated purchasing seed of some species from specialized native seed providers (Western Native Seed Company [Coaldale, Colorado, USA], WindRiver Seed [Manderson, Wyoming, USA], and Prairie Moon Nursery [Winona, Minnesota, USA]). Two varieties of invasive *Agropyron cristatum* were also included. Seed was surface sterilized, and seedlings were established in growth chambers with varying conditions determined to best facilitate their individual germination. Seeds were sown into once autoclaved vermiculite in aluminum trays. Since seedlings established at varying times, seedlings were transplanted from February 8 through March 3, 2014. [Note: AMF viability remained high as indicated by *Allium cemuum* (Fig. S1) which was one of the last species transplanted].

To establish the mycorrhizal growth response experiment, each species was grown in ten pots inoculated with AMF and ten pots inoculated with the (sham) control (e.g., Koide & Li, 1989). Seedlings of each plant species were planted into Deepots™ (500 ml, Steuwe and Sons, Tangent, Oregon, USA) mostly filled (~90% of pot volume, ca. 500 ml) with once autoclaved (2 hr) field soil (Eapa loam). The field soil was collected in 2013 from an upland plain in northern mixed‐grass prairie, mechanically sieved through a 6.5‐mm screen. The grassland soil was a well‐drained loam with pH of 7.3, 1.3% organic matter, 7.3 ppm NO_3_‐N, 16 ppm P [Bray test 1], and 306 ppm potassium. The soil series has a B horizon containing carbonates which may immobilize P (e.g., Lajtha & Schlesinger, 1988).

Deepots™ were filled with 59 ml of once autoclaved gravel (to obstruct drain holes), 340 ml of once autoclaved field soil, 50 ml of inoculum (AMF or control), and topped with once autoclaved field soil. All stages of the experiments were conducted in a growth room facility at Fort Keogh Livestock and Range Research Laboratory, which was modeled after the facility at the International Culture Collection of (Vesicular) Arbuscular Mycorrhizal Fungi at West Virginia University. The space housed three identically designed grow benches, each with two 400 W high‐output sodium vapor lights (photosynthetically active radiation average = 492 ± 57 [95% CI] μmol × m^−2^ × s^−1^). The growth room was maintained on a 12‐hr day and night temperature cycle with day temperature maintained between 20.0 and 22.2°C and night temperatures maintained between 16.7 and 18.9°C. These temperature ranges were similar to those used by Wilson and Hartnett (1998) while conducting their greenhouse experiment. Pots were carefully watered by hand to avoid splash transfer among pots. Pots were provided with a small quantity of reverse osmosis water every other day to ensure a relatively stable soil moisture environment throughout the week and one main watering event per week to ensure pots returned to field capacity at least once per week. Deepot™ racks were randomized weekly.

After 100 days since the time of planting, the plants were harvested. As we were also interested in determining the palatability of the shoots for another study, shoot material was freeze‐dried (Freezone 6, Labconco, Kansas City, MO, USA) to constant weight and weighed. We did not collect data for pots with dead plants. We retained two species in the study with moderate levels of mortality *Andropogon gerardii* (45%) and *Panicum virgatum* (30%), and mortality was evenly distributed across the two inoculant treatments (data not shown). Previous trials indicated estimates of root biomass could be inflated by residual clay soil that remained attached to washed root samples. To overcome this, roots were separated from the soil by hand, dried to constant mass, weighed, combusted at 450°C for 4 hr, cooled to 60°C, and reweighed to determine the proportion of ash‐free dry mass (AFDM) after combusting the samples. Total plant biomass was then determined by summing the shoot dry mass with root weight (root dry mass × proportion AFDM). Plant biomass data for analyses included total biomass, shoot mass, and root mass ratio (root mass × total biomass^−1^).

To assess the infectivity of AMF inoculum, we quantified root infection by AMF (%) on a subset of species (McGonigle, Miller, Evans, Fairchild, & Swan, 1990) at the conclusion of the mycorrhizal growth response experiment. We also sequenced the DNA of pooled root samples and identified 11 AMF virtual taxa (VT) based on MaarjAM (Öpik et al., 2010). The two most abundant taxa matched with *Funneliformis mosseae* (VT67) and *Claroideoglomus* (VT402), whereas the rest matched with *Glomus* (VTs 130, 135, 140, 165, 172, 332, 416), *Claroideoglomus* (VT193) and *Acaulospora* (VT21). For a brief explanation of methods used for DNA extraction, amplification, sequencing, and bioinformatics, please see Appendix S1 in Supporting Information.

Methods information for the Central Plains mycorrhizal responsiveness experiment can be found in Wilson and Hartnett (1998).

### 2.2 Phylogenetic reconstruction

To analyze data while simultaneously controlling for the effects of shared ancestry among plant species, we used Reinhart et al.'s (2012) phylogeny (represented as a phylogram) for the Central Plains dataset, and also constructed a new phylogeny for the Northern Plains' taxa using gene sequence data. To construct the latter phylogeny, we searched the GenBank database for four gene sequences often used to construct angiosperm phylogenies: *matK*,* rbcL*,* ITS1*, and *trnL*. Multiple gene sequences are necessary to differentiate plant species because some sequences represent conservative coding regions (e.g., *rbcL*) and others represent more rapidly evolving portions (e.g., *matK*) (Reinhart et al., 2012). When a sequence was not available for the exact same species for a marker, we used sequences available for species of the same genus, and when possible of species also native to North America. This was performed for eight of 68 species. We included one representative from an early diverging angiosperm lineage as an outgroup species, *Magnolia grandiflora* (L.). Sequences of these 69 species were aligned using MUSCLE version 3.7 (Edgar, 2004). We identified the best‐fit maximum‐likelihood models of nucleotide substitution for each gene using Akaike Information Criterion using MrModeltest version 2.3 (Nylander, 2004).

Using the concatenated sequence alignments, we performed a partitioned Bayesian Inference, estimating the posterior probability distribution of all possible phylogenies using a Markov Chain Monte Carlo algorithm (i.e., Metropolis algorithm) implemented in MrBayes version 3.1.2 (Huelsenbeck & Ronquist, 2001). To guide the search, we created a topological constraint tree from a published super tree (R20100428; Angiosperm Phylogeny Group, 2009), pruned to the taxa used in our experiment using Phylomatic (Webb & Donoghue, 2004). Two independent Markov Chains were run, each with four heated chains for 5 million generations. The final average standard deviation of split frequencies was 0.002, indicating good convergence of the two independent runs. We sampled runs every 500 generations and used a burnin of 5,000 trees to generate a majority rule consensus phylogeny. We transformed this, and the prior Central Plains, phylograms using penalized likelihood and maximum likelihood with “chronos” in APE version 3.2 in R. In order to create a time calibrated tree with branch lengths in millions of years (MYA), calibration points for seven nodes based on Bell, Soltis, and Soltis (2010) were used: Asteraceae (31–48 MYA), Plantaginaceae (34–51), Caryophyllales (79–96), Fabaceae (47–75), Malpighiales (88–91), Brassicaceae (21–42), and Poaceae (17–38). The newick‐formatted phylograms are provided in Appendix S2.

### 2.3 Statistical analysis

We tested the effects of treatment (AMF vs. control) on response variables for each plant species with two‐sample *t* tests that assumed either equal or unequal variances using Proc ttest in SAS version 9.4 (SAS Institute, Inc., Cary, NC, USA) as originally done by Wilson and Hartnett (1998).

We expressed plant growth response to AMF inoculation using two common effect size metrics: log response ratio (LRR; ln[response variable with AMF/response variable without AMF] and standardized effect size (SES; standardized mean difference between the AMF inoculated and control, relative to the pooled population variances). In these formulations, positive values indicate that plant growth was stimulated by AMF, whereas negative values indicate that plant growth was suppressed by AMF. Mycorrhizal responsiveness was calculated for plant total biomass and shoot biomass for the Northern Plains dataset and total biomass for the Central Plains dataset using metafor R‐library with measure = “ROM” or “SMDH.”

While Bunn et al. (2015) reported no appreciable difference between invasive and native taxa, they interpreted the magnitude of responses for native taxa tended to be greater than those for invasive taxa. To help address this, we tested whether the variances of SES (Northern Plains) and LRR (Central Plains) varied by invasive status with *F* tests for equality of variances using the “var.test” function in R.

To test whether mycorrhizal responsiveness (LRR or SES) varied by grassland plant invasive status, we conducted a phylogenetic ANOVA (phylo‐ANOVA hereafter) (Garland, Dickerman, Janis, & Jones, 1993) with the phylANOVA function implemented in the R‐package phytools (Revell, 2012). We also tested whether mycorrhizal responsiveness and invasive status were phylogenetically conserved, by calculating a phylogenetic signal. Phylogenetic signal of continuous data (mycorrhizal responsiveness) was assessed with Abouheif's C_mean_ (function adouheif.moran with method = “oriAbouheif” in adephylo R‐library) and Blomberg's *K* (function phylosig with method = “*K*” in phytools R‐library) and categorical data (invasive status) with the *D* statistic (function phylo.d in caper R‐library). For the *K* statistic, values close to 1 indicate that traits follow a Brownian motion (BM) model of evolution, *K* > 1 indicate that taxa are more similar than expected (trait conservatism), and when *K* < 1, closely related taxa are more dissimilar than expected (trait divergence) under BM evolution (Blomberg, Garland, & Ives, 2003). Because Blomberg's *K* relies on branch length information, we also used Abouheif's C_mean_ test, which provides an estimate of phylogenetic signal in a quantitative trait solely based on tree topology (Abouheif, 1999). For the *D* statistic, values close to 0 mean that the trait follows a Brownian motion model of evolution, whereas when *D* = ~1, the trait evolves randomly with respect to the phylogeny. When *D* < 0, then the trait is conserved relative to a BM model of evolution, whereas when *D* > 1, the trait is more divergent than expected.

## 3 RESULTS

**Figure 1:**
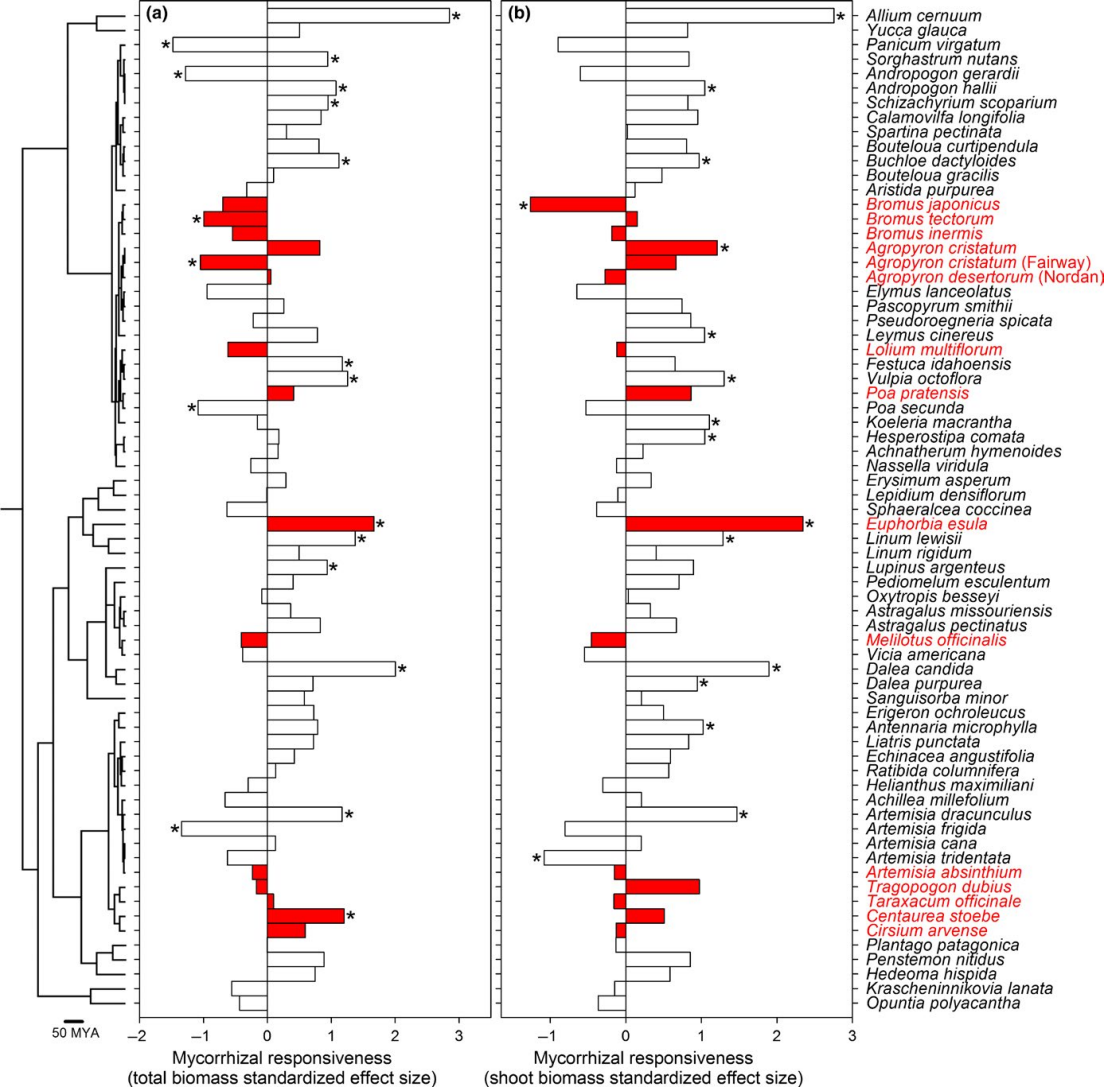
Molecular phylogeny and mycorrhizal responsiveness measures for 68 grassland plant species from the Northern Plains. Taxa labels and bars shaded red indicate taxa classified as invasive. Effect sizes were based on total plant biomass (a) and shoot biomass (b). Positive standardized effect size measurements indicate plants were larger with arbuscular mycorrhizal fungi than sham‐inoculant controls. We tested whether plants inoculated with AMF had significantly different biomass from controls by two‐sample *t*‐test (*p ≤* *.05)

**Figure 2:**
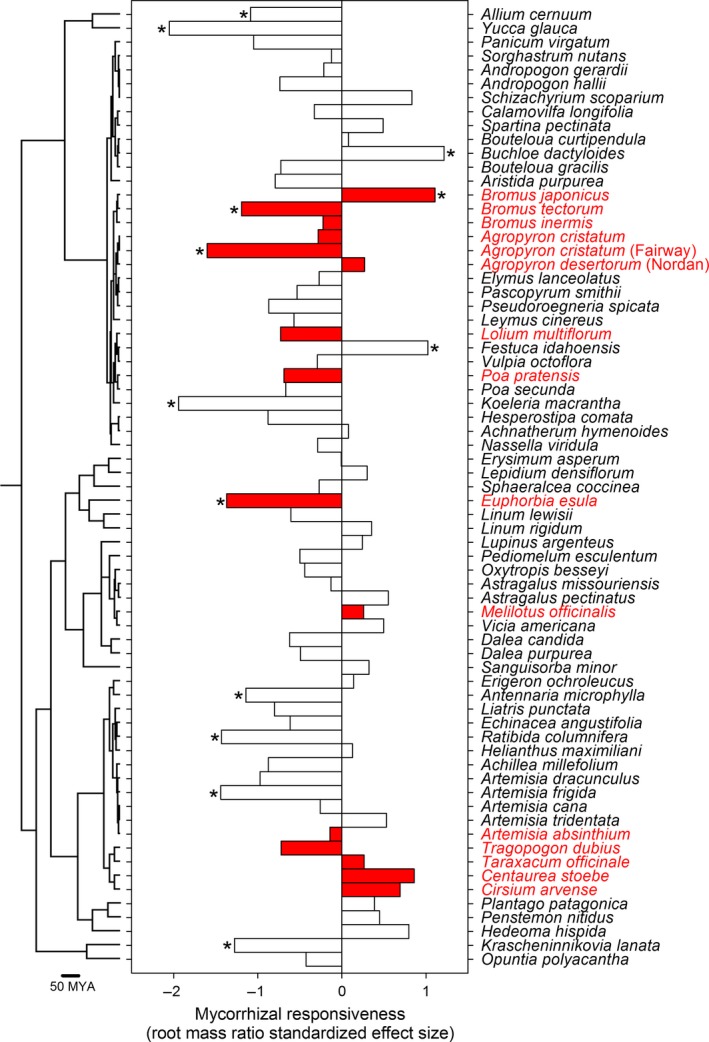
Molecular phylogeny and plant allometry (i.e., root mass ratio) for 68 plant species when grown with arbuscular mycorrhizal fungi (AMF) versus sham‐inoculant controls. Taxa labels and bars shaded red were classified as invasive. Negative standardized effect size measurements indicate that plants grown in soil with AMF allocated more resources to shoot than root biomass. We tested whether plants inoculated with AMF had significantly different root mass ratios from controls by two‐sample *t*‐test (*p ≤* *.05)

For the Northern Plains dataset, we confirmed that the roots of plants grown in pots with AMF inoculant were colonized by AMF (% root length colonized; range 4%–87%) while roots in controls (sham‐inoculant) were not (Fig. S1). Most Northern Plains species (71%–80%; three response variables) did not respond to inoculation with AMF and had similar total and shoot biomass and root mass ratio when grown with AMF versus sham‐inoculant controls. Nineteen percent of plants (13 of 69 comparisons) had greater total biomass when grown with AMF than controls (Figure 1a), whereas seven taxa had lower total biomass when grown with AMF relative to the sham‐inoculant control (Figure 1a). Eleven taxa allocated less biomass to roots than shoots when grown with AMF compared to controls (Figure 2). However, three taxa (*Bromus tectorum*,* Buchloe dactyloides*, and *Festuca idahoensis*) allocated appreciably more to roots than shoots when grown with AMF (Figure 2). The SES effect sizes for invasive and noninvasive taxa (Figure 1) also had equal variances (total biomass, *F*_14,53_ = 0.93, *p* = .93; shoot biomass, *F*_14,53_ = 1.40, *p* = 0.37).

Most species (71%–79%) classified as invasive for the Northern Plains dataset did not grow appreciably larger or smaller with AMF than controls (Figure 1). Two prominent invasive forbs (*Centaurea stoebe* and *Euphorbia esula*) produced greater total biomass when grown in soil with AMF than control soils (Figure 1a). One invasive grass (*Agropyron cristatum*) and forb (*E. esula*) produced greater shoot biomass in pots with AMF than controls (Figure 1b). Two invasive grass species (*A. cristatum* [Fairway] and *B. tectorum*), however, had lower total biomass in AMF inoculated vs. control pots (Figure 1a), and one invasive grass (*B. japonicus*) had lower shoot biomass in AMF inoculated vs. control pots (Figure 1b).

**Figure 3:**
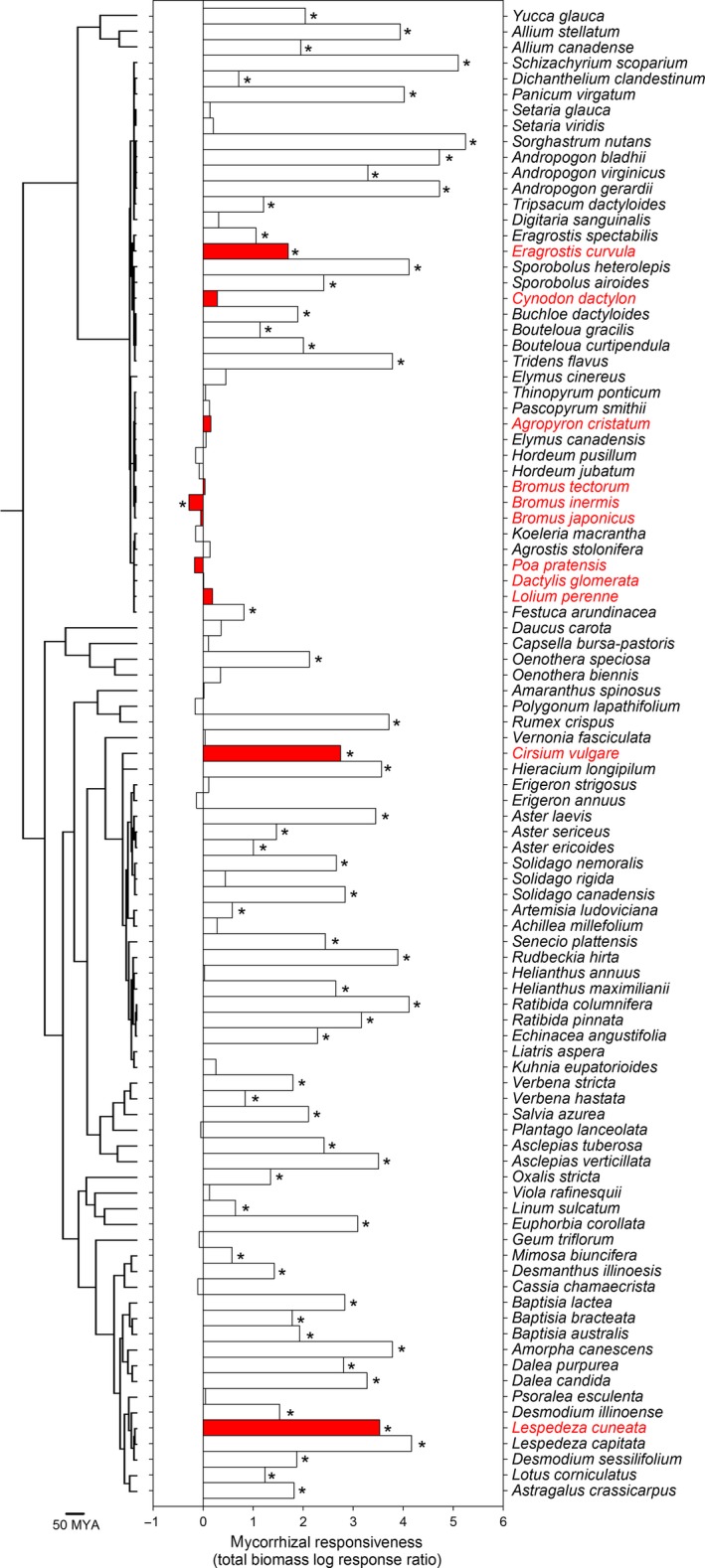
Molecular phylogeny and mycorrhizal responsiveness for 95 grassland plant species from the Central Plains (Wilson & Hartnett, 1998). Taxa labels and bars shaded red indicate taxa classified as invasive. Effect sizes were based on log response ratios (ln[mean biomass of plants with AMF/mean biomass of plants without AMF]) of total plant biomass data. Positive log response ratios indicate plants were larger with arbuscular mycorrhizal fungi than controls. Wilson and Hartnett (1998) tested whether plants inoculated with AMF had significantly different biomass from controls by two‐sample *t*‐test (*p ≤* *.05)

In the Central Plains dataset, by contrast, 61% (58 of 95) of grassland plant species had greater total biomass with AMF than controls (Figure 3) (Wilson & Hartnett, 1998). Only one taxon (*B. inermis*) had reduced growth in AMF inoculated vs. control soils (Figure 3). For the Central Plains portion, most species (64%) classified as invasive did not grow appreciably larger or smaller with AMF than controls (Figure 3). Three (*Cirsium vulgare*,* Eragrostis spectabilis*, and *Lespedeza cuneata*) of eleven invasive species had greater total biomass with AMF than without, and one invasive grass (*B. inermis*) had less total biomass with AMF than without (Figure 3). The LRR effect sizes for invasive and noninvasive taxa (Figure 3) had equal variances (total biomass, *F*_10,83_ = 0.71, *p* = 0.58).

We found no evidence (*p *≥* *.16) that plants classified as invasive interacted differently with AMF than noninvasive taxa for the Northern and Central Plains datasets while controlling for phylogenetic similarity (Table 1). Analyses for the Northern Plains portion included two effect size measurements (i.e., log response ratio and standardized effect size) for each of three response variables (i.e., total biomass, shoot biomass, and root mass ratio) (Table 1). The Central Plains dataset was more limited in terms of effect size measurements but there were still no appreciable differences among invasive and noninvasive taxa (Table 1).

**Table 1:** Phylogenetic ANOVA results for differences in effect size (log response ratio [LRR] and standardized effect size [SES]) by invasive status (invasive vs. noninvasive) of grassland plants from the Northern and Central Plains. Effect sizes differentiate plant responses when grown with arbuscular mycorrhizal fungi versus a sham‐inoculant control

Biomass data	Effect size	Northern Plains	Central Plains^a^
Total biomass	LRR	*F* = 1.33, *p *=* *.23	*F* = 3.75, *p *=* *.43
	SES	*F* = 2.02, *p *=* *.16	No data
Shoot biomass	LRR	*F* = 1.31, *p *=* *.24	No data
	SES	*F* = 1.28, *p *=* *.23	No data
Root mass ratio	LRR	*F* = 0.14, *p *=* *.72	No data
	SES	*F* = 0.17, *p *=* *.66	No data

a Data from Wilson and Hartnett (1998).

Invasive status was nonrandomly distributed over the phylogeny of taxa from the Northern Plains (*D*‐statistic = 0.31, *p *<* *.001; Figure 1) and Central Plains (*D*‐statistic = 0.32, *p *<* *.001; Figure 3) and followed a Brownian model of evolution in both the Northern (*p* =  .161) and Central (*p* =  .176) Plains. Invasive taxa were primarily from two plant clades (i.e., Asteraceae, Poaceae), which contained a mix of invasive and noninvasive taxa. Overall, we observed little evidence that effects of AMF on plants from the Northern Plains were strongly dependent on phylogeny. For the Northern Plains dataset, we did not detect evidence that AMF responsiveness was phylogenetically conserved using log response ratios for total biomass (Abouheif's C_mean_, *p *=* *.24, *K* statistic, *K *=* *0.02, *p *=* *.90) and shoot biomass (Abouheif's C_mean_, *p *=* *.20, *K* statistic, *K *=* *0.02, *p *=* *.82) or standardized effect sizes for total biomass (Abouheif's C_mean_, *p *=* *.06, *K* statistic, *K *=* *0.03, *p *=* *.64; Figure 1), shoot biomass (Abouheif's C_mean_, *p *=* *.24, *K* statistic, *K *=* *0.03, *p *=* *.75), and root mass ratios (Abouheif's C_mean_, *p *=* *.13, *K* statistic, *K *=* *0.04, *p *=* *.38; Figure 2). There was, however, a phylogenetical signal for log response ratios of root mass ratio data (Abouheif's C_mean_, *p *=* *.009, *K* statistic, *K *=* *0.07, *p *=* *.03). In contrast to the Northern Plains dataset, we observed a phylogenetic signal for log response ratios of total biomass data (Abouheif's C_mean_, *p *=* *.001, *K* statistic, *K *=* *0.16, *p *=* *.004; Figure 3) for the Central Plains. In both datasets *K* < 1, indicating that related taxa tended to have more divergent responses to AMF than expected.

## 4 DISCUSSION

Overall, using phylogenetically controlled statistical tests, we found no evidence that invasive grassland plant species responded differently to inoculation with AMF relative to other plant taxa in either the Northern or Central Plains dataset. Plant invasive status in both datasets was nonrandomly distributed across the phylogeny; invasive plants were disproportionally from two plant families (Asteraceae and Poaceae; Figures 1, 2, 3). Although there was a significant phylogenetic signal for mycorrhizal responsiveness with the Central Plains dataset (Figure 3), the overall pattern (*K* < 1) indicated that closely related taxa were more likely to differ than expected from a Brownian random walk model of evolution (Ackerly, 2009). Others have also reported that invasive plant taxa occur predominantly in a few plant clades (Lim, Crawley, de Vere, Rich, & Savolainen, 2014) and that mycorrhizal responsiveness exhibited greater divergence than convergence (*K* < 1) for grassland taxa (Anacker et al., 2014). The relatively weak effects of phylogeny, and the finding that mycorrhizal responsiveness is more likely to be divergent than conserved suggest that predicting mycorrhizal responsiveness in different ecosystems may be enhanced by considering key functional traits associated with root function or key genes related to mycorrhizal interactions, rather than phylogenies constructed with neutral molecular markers (e.g., *matK*,* rbcL*) used to identify plants.

Our findings are consistent with a recent meta‐analysis that compared invasive non‐natives to natives, albeit without the inclusion of phylogenetic information (Bunn et al., 2015), but contrary to a prior analysis of the Central Plains dataset (Fig. 3 in Pringle et al., 2009). In the latter case, inclusion of phylogenetic information helped to partition variance due to invasive status and phylogenetic relatedness which was not possible by Pringle et al. (2009). When phylogenetic relatedness was not controlled (i.e., standard ANOVA), results for the Central Plains dataset continued to provide marginally significant evidence that invasive taxa were less responsive to AMF (ANOVA, *F*_1,93_ = 3.745, *p *=* *.056) similar to related comparisons by Pringle et al. (2009). Phylogenetic analyses, however, indicated a nonrandom phylogenetic signal for AMF responsiveness with the Central Plains dataset and enabled us to differentiate whether AMF responsiveness varied by invasive status and/or phylogeny. Nevertheless, phylogeny was a weak overall predictor of mycorrhizal responsiveness for the Central Plains dataset (Reinhart et al., 2012) partly because related taxa tended to have greater trait divergence (*K* < 1) than convergence (*K* > 1). Trait divergence may be a result of related (and neighboring) plants associating with divergent AMF communities (Reinhart & Anacker, 2014; Veresoglou & Rillig, 2014) and/or other factors that contribute to niche partitioning among related plant species.

The Northern Plains region is known to support some of North America's most invasive plant taxa, including *Bromus tectorum*,* Centaurea stoebe*, and *Euphorbia esula*. Among the 14 invasive species in the Northern Plains dataset, three (*Agropyron cristatum*,* C. stoebe*, and *E. esula*) responded positively to AMF and three (*A. cristatum* [Fairway], *B. japonicus*, and *B. tectorum*) responded negatively to AMF. These divergent responses likely contributed to the lack of differences between invasive and other taxa. These findings support the hypothesis that a multitude of factors (e.g., anthropogenic disturbance, plant–microbe interactions, traits) contribute to invasiveness (Hierro et al., 2016; e.g., Maron et al., 2013; Müller, Horstmeyer, Rönneburg, van Kleunen, & Dawson, 2016).

It is not too surprising that on average invasive taxa are not less responsive to AMF as invasiveness may be facilitated by either decreased or increased mycorrhizal responsiveness. For example, the invasive grass *B. tectorum* is known to reduce AMF abundance (Busby, Stromberger, Rodriguez, Gebhart, & Paschke, 2013; Lekberg et al., 2013) and responded either negatively or neutrally to AMF (Figures 1 and 3). Other invasive grasses (*A. cristatum*,* B*. *inermis*) also appear capable of reducing AMF diversity (Jordan, Aldrich‐Wolfe, Huerd, Larson, & Muehlbauer, 2012) and had varying responses to AMF (Figures 1 and 3). Some invasive plants are thought to degrade AMF communities (Hale & Kalisz, 2012; Pakpour & Klironomos, 2015; Stinson et al., 2006; Vogelsang & Bever, 2009; Zhang et al., 2010) which may contribute to soil legacy effects that negatively impact the resilience of resident plant communities (Jordan, Larson, & Huerd, 2008; Perkins & Nowak, 2012, 2013). In contrast, some invasive forbs (i.e., *C. stoebe* and *E. esula*) increased the abundance and diversity of AMF communities (Lekberg et al., 2013), responded positively to AMF (Figure 1), and AMF enhanced the invasiveness of *C. stoebe* (Callaway, Mahall, Wicks, Pankey, & Zabinski, 2003; Marler et al., 1999). Therefore, mycorrhizal responsiveness facilitates the invasion of particular plant species, but weak mycorrhizal responsiveness is seemingly not a general mechanism of plant invasion.

Dominant native plants in the Northern Plains tended to be unresponsive to AMF. Specifically, four of the most prominent plants in the Northern Plains (*Artemisia tridentata*,* Bouteloua gracilis*,* Hesperostipa comata*, and *Pascopyrum smithii*) routinely associate with AMF (Bethlenfalvay & Dakessian, 1984; Carter, Smith, White, & Serpe, 2014; Reinhart & Anacker, 2014), but we found that only *H. comata* increased shoot biomass with AMF (Figure 1b). *Artemisia tridentata* actually had less shoot biomass with AMF than without. Other studies reported no effect of AMF on *A. tridentata* during mine reclamation (Biondini, Bonham, & Redente, 1985; Stahl, Williams, & Christensen, 1988), restoration postfire (review by Dettweiler‐Robinson et al., 2013), and a controlled mycorrhizal responsiveness test (Busby et al., 2011). Yet, other experiments indicated that *A*. *tridentata* seedlings had increased drought tolerance (Stahl, Schuman, Frost, & Williams, 1998), biomass (Stahl et al., 1988), and survival [fall but not spring transplants] with AMF (Davidson, 2015). Prominent congeners responded both positively (*A. dracunculus*) and negatively (*A. frigida*) to AMF. *Bouteloua gracilis* and *P. smithii* were also used in the Central Plains dataset, and *B. gracilis* plants were appreciably larger with AMF (Figure 3).

Although not a study aim, we are compelled to mention the apparent differences in AMF responsiveness for the two Plains ecoregions (Figure 1 vs. 3). Specifically, 61% of plants in the Central Plains were larger with AMF than without. Yet, appreciable positive effects of AMF on total plant biomass and shoot biomass were detected for only 28% of grassland plants in the Northern Plains. This apparent difference between Plains regions is intriguing, but we cannot reconcile whether such a difference is due to important methodological differences between studies or important ecological differences between Plains regions. There is a small body of literature suggesting ecological differences are possible—agronomic plants in calcareous soils were often less responsive to AMF (Li, Zhu, Marschner, Smith, & Smith, 2005; Zhu, Smith, & Smith, 2003). Calcium carbonates (which are prevalent in dryland soils like the Northern Plains) are known to reduce the bioavailability of phosphorus (e.g., Belnap, 2011) and may impact mycorrhizal mutualisms. Further testing is necessary to determine whether these regions truly have divergent interactions with AMF and the factors contributing to such differences.

In conclusion, despite substantial differences in functional characteristics, taxa sampled, and growth conditions for the experiments, we found no evidence to support the hypothesis that invasive taxa differ from native taxa in the degree of mycorrhizal responsiveness. Although we considered interspecific differences in the present analysis (i.e., a single genotype represented most plant species), others have noted that plant–AMF interactions vary by cultivars of invasive taxa like *A. cristatum* (Jun & Allen, 1991; Figure 1). Genotypic and geographical variation in plant–AMF interactions likely complicates formulation of robust generalizations for taxa with broad distributions. For instance, other plant–AMF interactions are conceivable with other pairings of AMF communities (species and genotypes), plant genotypes (e.g., Klironomos, 2003; Stahl & Smith, 1984), and growth conditions. Additional experiments that incorporate population and genotypic variability are necessary to quantify general effects of soil microbes across a plant's range (Callaway, Bedmar, Reinhart, Silvan, & Klironomos, 2011; Reinhart, Royo, van der Putten, & Clay, 2005; Wagner, Antunes, Ristow, Lechner, & Hensen, 2011).

## CONFLICT OF INTEREST

None declared.
