# Supplementary material for: Does responsiveness to arbuscular mycorrhizal fungi depend on plant invasive status?
Source: Ecol Evol. 2017 Jul 10;7(16):6482–92. doi: 10.1002/ece3.3226 (PMC5574787; doi:10.1002/ece3.3226)
Supplement: Supplementary file 1 [file ECE3-7-6482-s001.docx]

**Supplemental information**

**Fig. S1.** Mean percent root colonization

Figure S1. Mean percent root colonization (±95% CI) by arbuscular mycorrhizal fungi (AMF) of a sample of grassland plant species that were relatively responsive to AMF. Confidence intervals were determined using Pearson-Klopper method.

Fig. S1
