# Supplementary material for: Does responsiveness to arbuscular mycorrhizal fungi depend on plant invasive status?
Source: Ecol Evol. 2017 Jul 10;7(16):6482–92. doi: 10.1002/ece3.3226 (PMC5574787; doi:10.1002/ece3.3226)
Supplement: Supplementary file 2 [file ECE3-7-6482-s002.docx]

**Supplemental information**

**Appendix S1.** Methods for molecular characterization of AMF inocula.

Molecular characterization of AMF inocula

DNA was extracted from 25 mg of freeze-dried roots with the PowerPlant**^®^** Pro DNA isolation kit (MoBio Laboratories, Inc. Solana Beach, CA) following the manufacturer’s instructions. We used a two-step PCR protocol to generate amplicon libraries. For PCR1, we used the universal eukaryotic primer WANDA (SI from Dumbrell *et al.* 2011) and the Glomeromycota specific primer AML2 (Lee, Lee & Young 2008). External to the primers, we added 22 bp Fluidigm universal tags (Fluidigm Inc. San Francisco, CA, USA), allowing adhesion of PCR2 primers to amplicons produced by PCR1 reactions. The universal tag CS1 was added to the forward primer complex, and the universal tag CS2 was added to the reverse primer complex. PCR1 reactions were carried out in 25 µL volumes with 1 µL of DNA template, 5x GoTaq Buffer, 25 mM MgCl_2_, 20 mg/ml BSA, 10mM dNTP mix, 10 µM forward primer, and 10 µM reverse primer. Thermocycler conditions were: 95°C for 2 min; 30 cycles of 95°C for 1 min, 54°C for 1 min, 72°C for 1 min; and 72°C for 10 min, on a Bio Rad C1000 thermal cycler.

In our PCR2 reaction, we flanked amplicons generated from PCR1 with 8 bp barcode adaptors and Illumina flowcell adaptors (P5 and P7, Illumina Inc., San Diego, CA, USA). PCR2 primer complexes consisted of the same CS1 and CS2 Fluidigm tags, which allow for adhesion of PCR2 primer complexes to PCR1 amplicons. Amplicons generated during PCR1 were diluted 15 fold prior to PCR2. PCR2 was carried out in 20 µL volume with 1 µL of diluted PCR1 product, 5x GoTaq Buffer, 25 mM MgCl_2_, 20 mg/ml BSA, 10mM dNTP mix, 2 µM forward primer, and 2 µM reverse primer. Thermocycler conditions were: 95°C for 1 min; 10 cycles of 95°C for 30 sec, 60°C for 30 sec, 68°C for 1 min; and 68°C for 5 min, on a Bio Rad C1000 thermal cycler. Successful amplification was verified by 1.5% agarose gel electrophoresis using a 100 bp DNA ladder (O’GeneRuler DNA Ladder, Thermo Scientific, USA) as a size standard. PCR products were then purified with magnetic beads (Agencourt AMPure, Beckman Coulter Genomics, USA), quantified and pooled to equimolar concentrations prior to sequencing.

Sequencing and bioinformatics

Sequencing was done at the Institute for Bioinformatics and Evolutionary Studies (iBEST) genomics resources core at the University of Idaho (<http://www.ibest.uidaho.edu/>; Moscow, ID, USA) on an Illumina MiSeq sequencing platform (Illumina Inc., San Diego, CA, USA). All bioinformatic analyses were conducted using ‘quantitative insights into microbial ecology’ (QIIME; Caporaso *et al.* 2010). We followed primary quality filtering parameters as recommended from Bokulich et al., (2013)with the exception of the maximum unacceptable Phred quality score, which we adjusted to 19. Of the 4,840 total reads*,* 2,446 reads were retained after quality filtering. Reads were then clustered into Operational Taxonomic Units (OTUs) at 97% sequence similarity and assigned to virtual taxa (VTs) using the MaarjAM database (Öpik *et al.* 2010) with the UCLUST algorithm (Edgar 2010). A match to a VT required ≥97% similarity and at least 90% coverage. Doubletons as well as taxonomically unassigned reads were removed from downstream analyses, which resulted in 2,435 total reads.

Acknowledgements

Sequencing performed by the IBEST Genomics Resources Core at the University of Idaho was supported in part by NIH COBRE grant P30GM103324.

Literature cited

Bokulich, N.A., Subramanian, S., Faith, J.J., Gevers, D., Gordon, J.I., Knight, R., Mills, D.A. & Caporaso, J.G. (2013) Quality-filtering vastly improves diversity estimates from Illumina amplicon sequencing. *Nat Meth,* **10,** 57-59.

Caporaso, J.G., Kuczynski, J., Stombaugh, J., Bittinger, K., Bushman, F.D., Costello, E.K., Fierer, N., Pena, A.G., Goodrich, J.K., Gordon, J.I., Huttley, G.A., Kelley, S.T., Knights, D., Koenig, J.E., Ley, R.E., Lozupone, C.A., McDonald, D., Muegge, B.D., Pirrung, M., Reeder, J., Sevinsky, J.R., Turnbaugh, P.J., Walters, W.A., Widmann, J., Yatsunenko, T., Zaneveld, J. & Knight, R. (2010) QIIME allows analysis of high-throughput community sequencing data. *Nat Meth,* **7,** 335-336.

Dumbrell, A.J., Ashton, P.D., Aziz, N., Feng, G., Nelson, M., Dytham, C., Fitter, A.H. & Helgason, T. (2011) Distinct seasonal assemblages of arbuscular mycorrhizal fungi revealed by massively parallel pyrosequencing. *New Phytologist,* **190,** 794-804.

Edgar, R.C. (2010) Search and clustering orders of magnitude faster than BLAST. *Bioinformatics,* **26,** 2460-2461.

Lee, J., Lee, S. & Young, J.P.W. (2008) Improved PCR primers for the detection and identification of arbuscular mycorrhizal fungi. *FEMS Microbiology Ecology,* **65,** 339-349.

Öpik, M., Vanatoa, A., Vanatoa, E., Moora, M., Davison, J., Kalwij, J.M., Reier, Ü. & Zobel, M. (2010) The online database MaarjAM reveals global and ecosystemic distribution patterns in arbuscular mycorrhizal fungi (Glomeromycota). *New Phytologist,* **188,** 223-241.
