# Supplementary material for: Does responsiveness to arbuscular mycorrhizal fungi depend on plant invasive status?
Source: Ecol Evol. 2017 Jul 10;7(16):6482–92. doi: 10.1002/ece3.3226 (PMC5574787; doi:10.1002/ece3.3226)
Supplement: Supplementary file 3 [file ECE3-7-6482-s003.docx]

**Supporting Information:**

**Appendix S2.** Phylogeny as a text string.

Species names are listed as six letter abbreviations.

Northern Plains phylogeny- (((Allcer:80.3549215,Yucgla:80.3549215):80.33255406,((((Panvir:12.44735889,(Sornut:2.976046542,((Andger:0.7953246127,Andhal:0.7953246127):0.5923911685,Schsco:1.387715781):1.588330761):9.471312348):6.762834546,((Callon:4.808285562,Spapec:4.808285562):7.432763262,((Boucur:3.693124452,Bucdac:3.693124452):1.284754224,Bougra:4.977878677):7.263170147):6.969144612):5.111819981,Aripur:24.32201342):11.37258157,((((((Brojap:4.423616738,Brotec:4.423616738):2.468652347,Broine:6.892269085):9.203226304,((Agrcri:4.265254959,Agrdes:4.265254959):6.463732934,((Elylan:3.208738853,Passmi:3.208738853):2.45799803,Psespi:5.666736883):5.06225101):5.366507496):1.873973326,Leycin:17.96946872):5.566651967,(((Lolmul:7.89854856,(Fesida:3.819475671,Vuloct:3.819475671):4.079072889):4.034272318,(Poapra:5.200962121,Poasec:5.200962121):6.731858758):5.907657981,Koemac:17.84047886):5.695641824):4.364560362,((Hescom:4.105160224,Achhym:4.105160224):20.91301163,Nasvir:25.01817185):2.882509191):7.793913947):124.9928806):127.3904432,((((((Eryasp:32.38775925,Lepden:32.38775925):43.009794,Sphcoc:75.39755326):42.4046047,(Eupesu:90.58924327,(Linlew:45.28797156,Linrig:45.28797156):45.30127172):27.21291468):42.48956266,((Luparg:53.82531292,(Pedesc:29.33873724,((Oxybes:12.21542999,(Astmis:3.391294541,Astpec:3.391294541):8.824135445):2.925098829,(Meloff:8.991504099,Vicame:8.991504099):6.149024716):14.19820842):24.48657568,(Dalcan:25.60496854,Dalpur:25.60496854):28.22034438):52.90166513,Sanmin:106.7269781):53.56474256):42.58986766,((((Erioch:16.11108623,Antmic:16.11108623):13.9297903,((Liapun:13.18905843,((Echang:7.952698471,Ratcol:7.952698471):1.743621606,Helmax:9.696320077):3.492738354):7.491438345,(Achmil:8.896953234,(Artdra:6.369283572,(Artfri:4.69575264,((Artcan:0.4020275761,Arttri:0.4020275761):2.339398116,Artabs:2.741425692):1.954326947):1.673530932):2.527669662):11.78354354):9.360379761):15.28781173,((Tradub:15.48899125,Taroff:15.48899125):14.74331211,(Censto:15.06190788,Cirarv:15.06190788):15.17039548):15.09638491):78.83806899,((Plapat:35.16171864,Pennit:35.16171864):44.22686606,Hedhis:79.38858469):44.77817257):78.71483101):42.45883901,(Kralan:95.47626297,Opupol:95.47626297):149.8641643):42.73749148);

Central Plains phylogeny-

(((((((((Astcra:18.09170956,Lotcor:18.09170956):14.29916099,(((Desses:7.36744025,(Lescap:4.158323791,Lescun:4.158323791):3.209116459):6.606010467,Desill:13.97345072):6.972661248,Psoesc:20.94611196):11.44475859):11.41416705,(((Dalcan:12.2676177,Dalpur:12.2676177):10.75348173,Amocan:23.02109943):15.59292537,((Bapaus:11.45928353,Bapbra:11.45928353):7.024494532,Baplac:18.48377806):20.13024674):5.191012807):18.20243876,(Cascha:32.46496651,(Dnthus:13.50558629,Mimbiu:13.50558629):18.95938022):29.54250986):49.38992036,Geutri:111.3973967):46.33058787,(((Eupcor:88,Linsul:88):25.68379416,Vioran:113.6837942):22.89222148,Oxastr:136.5760156):21.15196895):43.62029341,((((Ascver:51.26641817,Asctub:51.26641817):46.90830727,(Plalan:63.79074249,(Salazu:33.20181735,(Verhas:15.00028394,Verstr:15.00028394):18.20153341):30.58892514):34.38398295):35.88363094,((((((Kuhmac:7.170239067,Liaasp:7.170239067):6.349392897,(((Echang:1.113308939,(Ratpin:0.6455261686,Ratcol:0.6455261686):0.4677827701):7.404378536,(Helmax:6.100306774,Helann:6.100306774):2.4173807):1.631959053,Rudhir:10.14964653):3.369985436):8.005138774,Senpla:21.52477074):6.282170666,((Achmil:7.348416017,Artlud:7.348416017):15.58511075,((((Solcan:4.032915303,Solrig:4.032915303):1.638228288,Solnem:5.671143591):6.454468634,((Asteri:3.224089022,Astser:3.224089022):4.374402085,Astlae:7.598491107):4.527121119):2.137782006,(Eriann:7.483431174,Eristr:7.483431174):6.779963057):8.670132541):4.873414632):9.833775265,Hielon:37.64071667,Cirvul:37.64071667):46.40975912,Verfas:84.05047579):50.0078806):35.63919742,((Rumcri:44.07732753,Pollap:44.07732753):63.37056399,Amaspi:107.4478915):62.24966228):31.65072419):50.01650258,(((Oenbie:59.13829336,Oenspe:59.13829336):68.87833437,Capbur:128.0166277):66.75329214,Daucar:194.7699199):56.59486072):58.79477294,(((((((Fesaru:4.840619961,Lolper:4.840619961):0.8809626288,Dacglo:5.72158259):0.2641968614,Poapra:5.985779451):1.076442813,(Agrsto:4.780727431,Koemac:4.780727431):2.281494834):8.89812916,(((Brojap:3.194384809,(Broine:1.981736851,Brotec:1.981736851):1.212647958):1.704165758,(((Horjub:1.376474435,Horpus:1.376474435):1.459604212,Elycan:2.836078647):1.305107862,Agrcri:4.141186509,Agrsmi:4.141186509,Agrelo:4.141186509):0.7573640581):10.88608303,Elycin:15.7846336):0.1757178231):1.039648575,(((((Trifla:3.436447949,(Boucur:1.765847497,Bougra:1.765847497,Bucdac:1.765847497):1.670600452):2.05447993,Cyndac:5.49092788):0.2790155127,(Spoair:5.376856772,Spohet:5.376856772):0.39308662):5.947208587,(Eracur:4.903673947,Eraspe:4.903673947):6.813478032):3.547988059,(Digsan:6.473730879,(Tridac:3.210803936,(Andger:2.228358273,Andvir:2.228358273,Andbla:2.228358273,Sornut:2.228358273):0.9824456627):3.262926943,(Setvir:2.075437163,Setgla:2.075437163):4.398293716,(Panvir:4.691945891,Diccla:4.691945891):1.781784988,Andsco:6.473730879):8.791409159):1.734859962):144.2228393,((Allcan:47.23992497,Allste:47.23992497):52.35150903,Yucgla:99.591434):61.63140527):148.9367142);
